# The experience of physical activity and the transition to retirement: a systematic review and integrative synthesis of qualitative and quantitative evidence

**DOI:** 10.1186/1479-5868-9-97

**Published:** 2012-08-16

**Authors:** Inka Barnett, Cornelia Guell, David Ogilvie

**Affiliations:** 1 MRC Epidemiology Unit and UKCRC Centre for Diet and Activity Research (CEDAR), Box 296, Institute of Public Health, Forvie Site, Robinson Way, Cambridge, CB2 0SR, UK; 2 Faculty of Medical Sciences, University of West Indies, Bridgetown, Barbados

**Keywords:** Behaviour change, Physical activity, Retirement, Aged, Qualitative research

## Abstract

**Background:**

The transition to retirement has been recognised as a critical turning point for physical activity (PA). In an earlier systematic review of quantitative studies, retirement was found to be associated with an increase in recreational PA but with a decrease in PA among retirees from lower occupational groups. To gain a deeper understanding of the quantitative review findings, qualitative evidence on experiences of and views on PA around the transition to retirement was systematically reviewed and integrated with the quantitative review findings.

**Method:**

19 electronic databases were searched and reference lists were checked, citations tracked and journals hand-searched to identify qualitative studies on PA around the transition to retirement, published between January 1980 and August 2010 in any country or language. Independent quality appraisal, data extraction and evidence synthesis were carried out by two reviewers using a stepwise thematic approach. The qualitative findings were integrated with those of the existing quantitative systematic review using a parallel synthesis approach.

**Results:**

Five qualitative studies met the inclusion criteria. Three overarching themes emerged from the synthesis of these studies: these related to retirees’ broad concepts of PA, the motives for and the challenges to PA in retirement. Integrative synthesis of the qualitative findings with the quantitative evidence offered several potential explanations for why adults might engage in more recreational PA after the transition to retirement. These included expected health benefits, lifelong PA patterns, opportunities for socialising and personal challenges, and the desire for a new routine. A decrease in PA among retirees from lower occupational groups might be explained by a lack of time and a perceived low personal value of recreational PA.

**Conclusions:**

To encourage adoption and maintenance of PA after retirement, interventions should promote health-related and broader benefits of PA. Interventions for retirees from lower occupational groups should take account of busy post-retirement lifestyles and the low personal value that might be attributed to recreational PA. Future research should address predictors of maintenance of recreational PA after the transition to retirement, the broader benefits of PA, and barriers to PA among retirees from lower occupational groups.

## Background

Physical activity (PA) is beneficial for health and well-being in old age [1-6]. However, PA has been shown to decrease as adults become older [7-9]. The transition to old-age retirement has been recognised as a turning point for determining PA behaviour in later life [10]. Interventions targeted at this critical transition period might therefore be effective in promoting an active lifestyle in retirement.

We recently systematically reviewed quantitative evidence on changes in PA across the transition to old-age retirement [11]. Observational cross-sectional and longitudinal studies of free-living PA in all domains were included and no language or country restrictions were applied. To reflect the changing concept of retirement, we employed an exploratory approach to its definition and did not prescribe a specific age range. We synthesised the data using a semi-quantitative approach combined with a harvest plot for visualising the findings [12]. The review suggested that recreational PA increases with retirement, while no clear pattern emerged for overall PA. The impact of retirement on PA appeared to be moderated by socioeconomic status (SES), with PA (both recreational and overall) decreasing after retirement among adults from lower occupational groups but increasing or remaining the same among those retiring from higher occupational groups. A major limitation of the body of quantitative studies reviewed was the imprecise assessment of PA, which relied heavily on single questions or bespoke questionnaires of unknown validity.

The implications for future research and practice that can be drawn from the available quantitative studies are limited. Qualitative research might provide a deeper understanding of the underlying reasons for the changes in PA across the transition to retirement [13,14]. Therefore, the aims of this study were to systematically review qualitative research on experiences of and views on PA around the transition to retirement; to use this evidence to explain and interpret the findings of the quantitative review; and to integrate the qualitative and quantitative evidence in order to identify priorities for future research and interventions to promote the adoption and maintenance of PA after retirement.

## Methods

The qualitative review process followed standard methods for systematic reviews [15]. The qualitative synthesis was then used to aid explanation and interpretation of the findings of the existing quantitative review using an adaptation of the parallel synthesis approach as described in the Cochrane handbook for systematic reviews of intervention studies [16].

### Search for evidence

In August 2010 a comprehensive search was undertaken in 19 electronic databases relevant to PA, health and ageing: Abstracts of Social Gerontology, AMED, Anthropology Plus, ASSIA, BNI, CINAHL, Cochrane Library, EMBASE, ISI Web of Science, JSTOR, Medline, Physical Education Index, ProQuest Digital Dissertations, PsycInfo, Science Direct, Scopus, Social Service Abstracts, Sociological Abstracts and SportDiscus. The reference and citation lists of all included studies were checked and seven key peer-reviewed journals on ageing, health and PA were hand-searched. The search strategy included both broad-based free-text terms and thesaurus mapping to increase its sensitivity for detecting relevant qualitative evidence [17]. Search terms related to ‘physical activity’ and ‘retirement’ were used. The search strategy is available from the authors on request.

### Inclusion and exclusion criteria

Studies were included if they explored experiences of or views on free-living PA around the transition to old age retirement in community-dwelling individuals, were published after January 1980 and used established qualitative research methods. No country or language restrictions were applied. Journal articles and grey research literature (full conference papers, PhD theses and government reports) were eligible for inclusion.

Studies were excluded if they explored PA in old people without referring to the transition to retirement; if they were limited to physical capability or fitness; or if they focussed exclusively on ill-health or temporary retirees or retired athletes.

### Quality assessment

The quality assessment of qualitative research remains a challenge and is the subject of ongoing debate [18]. Some researchers [19,20] argue that the nature of qualitative research and the variety of methodological approaches may prohibit quality assessment based on a set of pre-defined validity criteria similar to those typically applied to the appraisal of quantitative evidence. Others [21,22] emphasise the need for quality assessment to identify robust qualitative research and facilitate its inclusion in systematic reviews. These advocates suggest using assessment tools that acknowledge the distinct underlying concepts of qualitative research [23,24].

For this review we used the 10-item checklist for qualitative studies developed by the Critical Appraisal Skills Programme (CASP) [25]. This checklist focuses on three key criteria of rigour, credibility and relevance without being excessively restrictive or prescriptive. We used the tool only to guide our appraisal of the studies and not to exclude any studies. Studies were independently appraised by two reviewers (IB, CG) who then discussed the findings.

### Data extraction and synthesis

A data extraction sheet was developed, piloted and used to collect information about the study objectives, geographical settings, sample size and selection and other details of the research methods. Data were extracted independently by two reviewers (IB, CG). A stepwise thematic approach was used for the synthesis of the qualitative evidence [26]. This approach involved an iterative process of reading and re-reading of the studies, the independent identification of themes concerning experiences of and views on PA around the transition to retirement by two reviewers (IB, CG), the comparison and consolidation of these initial themes into one list and finally a summary into overarching themes. We present participants’ quotations from the original studies to support these themes. For clarity, some of these quotes have been edited to provide a consistent style of quotation; ‘. . .’ indicates omissions from the original interview quotes by the papers’ authors, while ‘[…]’ indicates our own additional shortening of quotes. The qualitative findings were then juxtaposed with the quantitative evidence in order to enable a deeper understanding of and critical reflection upon the quantitative findings. Recommendations for future research and practice were drawn from this integrative synthesis.

## Results and discussion

### Search findings and study characteristics

**Figure 1:**
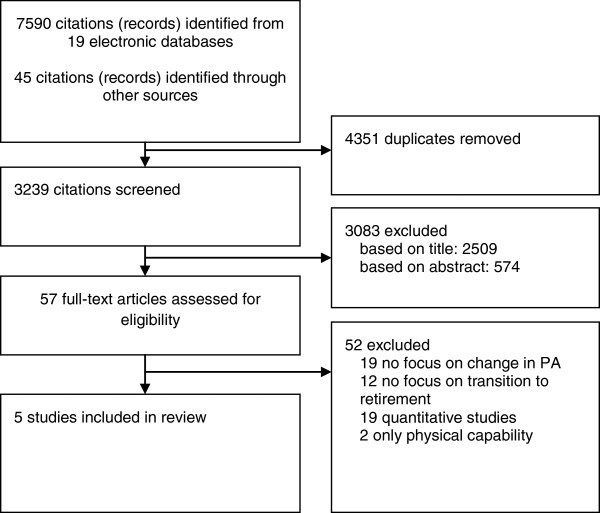
Review flowchart.

Of the 3,239 citations (excluding duplicates) identified, five qualitative studies [27-31] met the inclusion criteria (Figure 1). All studies were published after 2003, suggesting a relatively recent development of research interest in the topic. Table 1 summarises the methodological details of the included studies. All participants had been retired for between six months and 5.6 years, with three studies also including a small number of partly retired individuals. The majority of participants were from affluent socioeconomic backgrounds; only one study included participants from both higher and lower occupational groups and one study included only retired manual workers. All studies focussed on recreational PA; none had explicitly set out to explore overall PA. 

**Table 1:** Characteristics of the qualitative studies

**Author**	**Location**	**Aim**	**Sample**	**Design**	**Analysis**
Arkenford 2006 [27]	UK, community setting	To get a better understanding of facilitators of and barriers to sports participation in the recently retired	21 groups of 6-8 participants each, men and women aged 50-75	Focus groups	Thematic analysis
Beck et al. 2010 [28]	UK, community setting	To explore changes in PA behaviour in retirement using the perspective of self-determination theory	7 women and 4 men aged 57-65	In-depth interviews	Thematic analysis
Scanlon-Mogel & Roberto 2004 [29]	USA, members of a fitness centre	To explore life transitions and trajectories that encourage or hinder PA in older adults using a life course perspective	6 women and 9 men aged 65-75	In-depth interviews	Qualitative inquiry method (as defined by authors)
Strobl et al 2008 [30]	Germany, members of a fitness centre	To explore the role of PA in coping with the challenges of the transition to retirement	4 women and 6 men aged 59-69	In-depth interviews	Content analysis
Witcher 2007 [31]	Fogo Island, Canada, community setting	To explore older adults’ perceptions of recreational PA across their life course	5 women and 5 men aged 70-94	In-depth interviews	Inductive analysis

PA: physical activity.

### Quality assessment

All papers described the study design and research methods, but two studies provided insufficient details of the processes of sample selection [29,30] or data collection [27,30]. No information on ethical issues or research reflexivity was given in three studies [27,29,30]. Results were presented clearly and participants’ quotations were given in all studies. Two studies used member-checking to validate the findings from the analysis [28,31], one study included triangulation with field notes [29] and three studies reported the involvement of two or more researchers to independently describe and interpret the data [28,29,31].

### Synthesis of the qualitative findings

The following three overarching key themes were identified: the concept of PA; motives for PA in retirement; and challenges to PA in retirement.

#### The concept of PA

Retirees had broad concepts of PA that included recreational PA but also a wide range of domestic activities [27-31], as illustrated in the following two quotes: 

‘Cleaning as well, that can be quite aerobic . . . I think of it as exercise . . . I think well, I‘ve done some exercise today’ (Woman) [27]

‘You can’t do the garden without getting exercise. I do my hard work in the garden.’ (Man) [27]

Recreational PA and domestic PA were perceived as equally valuable for achieving sufficient PA levels and many retirees were convinced that they derived enough PA from their daily activities of living [27,29,31]: 

‘To me I’m active, but to a lot of people I’m boring and lazy. I walk and go to the shops, I go round the supermarket, I do housework and go down the garden and take the dog for a walk.’ (Woman) [27]

‘At the end of the day he spends two hours in the gym and I spend two hours out there digging, so I am getting as much exercise as he is.’ (Man) [27]

The concept of PA evolved over the life course [29]. In early adulthood participation in sports and organised exercise was perceived as the main source of PA. In middle and older adulthood definitions of PA expanded to include such activities as housework, caring responsibilities and gardening.

Retirees’ broad understandings of PA were acknowledged and critically reflected upon by the authors of two of the included studies [27,29]. In the other studies [28,30,31] researchers associated PA almost exclusively with recreational PA and either did not consider [30,31], or only marginally considered [28], the contribution of domestic and other domains of PA.

#### Motives for PA in retirement

Three motives for PA after the transition to retirement were identified: expected benefits for health and well-being; lifelong PA patterns; and broader benefits of PA.

##### Expected benefits for health and well-being

Participants described how the transition to retirement prompted a sudden awareness of their own ageing [30] and ‘the fact that the time is running out’ [27]. Recreational PA was adopted or increased to slow down physical and mental decline and to improve health and well-being [27-31]: 

‘When I retired, I realised that in order to stay ahead of the ageing process, I needed to engage in regular exercise.’ (Man) [29]

‘I have taken up physical activity in retirement ‘cause I wanna live as long a life as possible. And you know, while I can really […]’ (Man) [28]

The belief in the potential health benefits of PA could not ensure maintenance of PA, though, and relapse into inactivity was frequent [28].

Other participants were aware of the health benefits of PA, but this did not motivate them to adopt recreational PA themselves [27,28]: 

‘I know all the theory about keeping fit and healthy, and healthy heart and […] and all the rest of it. […] It’s not through lack of awareness it’s just lack of inclination.’ (Woman) [28]

‘Exercise is self-generating . . . if you are exercising your brain is better, your circulation is better, everything else is better. I’ve definitely noticed the difference in the last couple of years where I haven’t done anything — the brain is starting to fudge up a bit now!’ (Man) [27]

A few participants were not convinced that PA could be beneficial for their health, felt they were ‘too old to start’ [27,28] or feared injuries or pain from overexertion: 

‘If I go too far then my calf muscles will ache, I won’t be able to do anything — I will be in bed’ (Man) [27]

‘If you garden all day you end up with a bad back’ (Man) [27]

##### Lifelong PA patterns

Older adults believed that their lifelong participation in and enjoyment of recreational PA was a strong motivator for their continued and often increased participation after the transition to retirement [27-30]: 

‘Because if I hadn’t been active as a child and when I had kids at home, then I probably wouldn’t be now. I think that if you’re always active that you will stay active.’ (Woman) [29]

Similarly, a lifelong tendency for physical inactivity often persisted after retirement [27,28]: 

‘I never liked physical activity anyway . . . I’ve got along not doing it for 60 odd years’ (Woman) [28]

##### Broader benefits of PA

A new routine

Many participants felt the need to replace their working-day routine with new routines in retirement and intended to re-establish a sense of control and purpose in their lives [28,30]. For many retirees engagement in recreational PA formed part of or comprised the whole of the new routine. Others found new daily structures in less physically active pursuits such as book clubs and choir singing [28].

While not all retirees desired new routines in retirement, fixed schedules for participation in recreational PA were perceived as necessary to maintain PA and prevent procrastination [27-30]: 

‘I think it [taking exercise] was easier then [when I was at work] because now I’ve got all the time in the world. […]’ (Man; *additions in original quote*) [28]

Not planning recreational PA as a regular feature during the week often resulted in only occasional or ‘ad hoc’ PA [28].

##### A new personal challenge

Men in particular were eager to find new personal challenges. Participation in recreational PA could provide such a challenge [28,30]: 

‘During the time I’m at the gym the motivation is to try to challenge myself to do a little better […]. I try and do level 7 […], so there is always a challenge. […]’ (Man) [28]

Improved fitness, learning a new skill (e.g. martial arts or tap dancing [28]) or engaging in competitive team sports could help to foster the perceptions of independence or elevate self-worth and a sense of accomplishment [28,30]. These feelings were highly regarded among retirees who often feared becoming ‘useless’ [29], ‘invisible’ [27] or a burden to their families [28,30].

Some retirees described frustration with their diminishing physical abilities due to health problems and ageing and how this interfered with their capability to compete at desired levels. This often resulted in discontinuation of competitive sports [27,28]: 

‘You always remember how good you were and you will never aspire to those levels again so you think what is the point of even trying.’ (Man) [27]

##### An opportunity for social interactions

Women in particular missed the social interactions from their work environments and joined gyms and regular exercise classes to meet new people or friends, reduce loneliness, establish emotional support networks and convey a sense of belonging [27,28,30]: 

‘[…] I wanted to do something with another group of people so that you had the social side of it.’ (Woman) [28]

‘People will notice and it makes you feel nice . . . but it makes you feel . . . a bit special if people notice that you are not there.’ (Woman) [28]

Less physically active group activities could also provide opportunities for social interactions in retirement [28]. Male retirees acknowledged the social aspect as ‘a good by-product of taking exercise’ [27] but often perceived it as less important than women did.

For some retirees social interactions during recreational PA were a negative experience that let to discontinuation. Feelings of not being accepted or fitting into a group (for example, being ‘outnumbered [by women] twenty to one’ [28]) were cited.

#### Challenges to PA in retirement

Two challenges for PA in retirement emerged from the qualitative synthesis: lack of time, and the personal value of PA.

##### Lack of time

Contrary to general beliefs, many retirees described very busy lives:

‘ […] I never stop, from the time I get up, till I go to bed . . . around, from 7 o’clock in the morning, until around 11 in the night, I’m at something’ (Woman) [31]

‘Keeping busy’ [31] with domestic activities and family commitments was particularly important for retired manual workers and could compete for time with recreational PA [27,31]: 

‘When my father had a stroke a couple of years ago, it meant we couldn’t go anywhere. If something unexpected like this occurs, it takes up your time. So you can’t go to exercise classes with that all going on.’ (Man) [27]

Taking on voluntary or part-time work and less physically active leisure-time pursuits could also minimise the time available for recreational PA [27,28]. Some of these responsibilities or activities (such as the preference for less physically active pursuits) were long-term competitors for time. Others were described as short-term endeavours forming part of the process of adjustment to retirement (such as moving house or ‘bridging’ employment) [28,29] and participants who used to be physically active prior to retirement had plans to restart regular recreational PA once they were settled in retirement [28,29]: 

‘I’m sure we will finally establish a routine whereby I‘ll probably go back to Rosemary Conley [a popular diet and fitness regime], and then I’ll start my sort of exercise programme and swimming and stuff like that. […]’ (Woman; *our explanation added*) [28]

##### Personal value of PA

Inactive participants and those who had retired from manual occupations often placed a low personal value on recreational PA [28,31]: 

‘I don’t think my life depends upon walking, it’d be just a recreational thing.’ (Man) [28]

The low regard for recreational PA was often a deep-rooted belief among retired manual workers who had learned early on in their childhood that ‘it was better if you were doing work [than recreational PA]’ [31]. Throughout their lives, physical labour had been essential for subsistence; PA for recreational purposes was perceived as a waste of precious time. This conviction often persisted in retirement.

In contrast, purposeful or productive activities such as picking berries [31], fishing [31], carpentry [31], gardening [27,31], or housework [27,31] were highly valued and socially approved in retirement: 

‘Why jog? I just can’t see the point in it.’ ‘Why can’t they go and mow some OAP’s [old age pensioner’s] lawn instead.’ (Woman; our explanation added) [27]

### Integrative synthesis of qualitative and quantitative evidence

Table 2 summarises the integration of the quantitative and qualitative evidence. This began with the quantitative findings in the first column, which were then explained and interpreted with the help of the qualitative evidence presented in the second column. The final two columns illustrate some tentative recommendations for future research and practice derived from the combination of evidence. As none of the qualitative studies explored overall PA, only the main quantitative findings on recreational PA and on the effect of SES, in particularly the decrease in PA among retirees from lower occupational groups, were explored using this integrative method.

**Table 2:** Parallel synthesis of qualitative and quantitative evidence syntheses and recommendations for future research and practice

**Quantitative synthesis**	**Qualitative synthesis**	**Implications for research**	**Implications for practice**
Increase in recreational PA after the transition to retirement	Health properties of PA motivate adoption/increase of recreational PA but do not guarantee long-term maintenance	Assess long-term development of recreational PA patterns after retirement	Emphasise multiple benefits of recreational PA rather than focusing only on health benefits
		Examine predictors of and barriers to long-term maintenance of recreational PA after retirement	Offer support for long-term maintenance of recreational PA after retirement
	Lifelong PA habits influence recreational PA patterns after retirement	Assess predictors of change from lifelong inactivity to physically active lifestyle after retirement	Promote PA to previously inactive retirees Promote continuation of lifelong PA (e.g. provide opportunities for age-appropiate PA)
	Recreational PA provides a new daily routine	Examine the broader benefits of PA after retirement and their potential role in maintenance of PA	Promote recreational PA as a regular feature after retirement Offer regular exercise classes, walking groups, etc. designed for recently retired
	Recreational PA offers new personal challenges		Promote recreational PA as a new challenge (especially for men)
			Create opportunities for competitive recreational PA within the same age group/capability level
	Recreational PA provides opportunities for social interactions		Promote PA as an opportunity for socialising (especially for women)
			Create opportunities for social recreational PA for the recently retired
	Broad concept of PA that includes recreational and domestic PA	Self-reported PA assessments need to consider broad concept of PA	Raise awareness of necessary intensity levels and durations of PA after the transition to retirement and how to achieve them
		Use objective PA measurements to assess change in PA across the transition to retirement	
		Investigate the health benefits of domestic PA after the transition to retirement	
Decrease in PA after retirement among low SES	Lack of time for recreational PA	Further investigate barriers to PA in retirees from lower occupational groups	Raise awareness of benefits and acceptance of recreational PA among retirees from low SES and their communities
	Low perceived value of recreational PA and preference for productive/meaningful PA		Raise awareness of benefits of short bouts of PA across the day
			Promote purposeful physical activities (e.g. community projects, dog-walking)

PA; physical activity.

#### Quantitative review finding: increase in recreational PA after the transition to retirement

The qualitative evidence hinted at several reasons why adults might engage in more recreational PA after the transition to retirement. Expected health benefits motivated many recently retirees to adopt or increase PA, but might not ensure long-term adherence. These findings were reflected in earlier reviews in which the beliefs in the health outcomes of PA were positively associated with both the initiation of PA [32,33] and current PA behaviour [34-36] among middle-aged and older adults; the influence of these beliefs on maintenance of PA remained unclear [32,33]. Future longitudinal studies should investigate whether the newly acquired recreational PA patterns are maintained and identify predictors of and barriers to maintenance. Interventions to promote recreational PA in retirement should portray multiple benefits of PA rather than focusing only on health benefits in order to reach a wider audience and achieve long-term changes.

In the qualitative studies lifelong participation in recreational PA was perceived as a prerequisite for maintained and increased PA in retirement. Similarly, lifelong inactivity was seen to be carried on into later life. A positive association between PA in old age and previous engagement in PA was reported in two previous reviews [33,35] and Trost [36] and Schutzer [32] found that current PA behaviour was predicted by a history of PA in adulthood, but not in childhood. None of these reviews examined the long-term influences of lifelong inactivity. Future research should address how lifelong patterns of physical inactivity might be broken and how physical activity can most effectively be initiated and maintained after retirement. Interventions should aim to facilitate the continuation of lifelong PA and encourage the adoption of PA among inactive retirees.

The qualitative evidence suggested that many retirees initiated or increased recreational PA in retirement to satisfy needs that had previously been addressed through work. Regular participation in recreational PA could provide a new daily routine after retirement. Retired men sought new personal challenges in recreational PA, whereas women looked for opportunities to socialise. Only one previous review briefly addressed these broader motives for PA participation [34], highlighting the need for more research. Interventions should emphasise multiple potential benefits of PA participation and address gender-specific needs (see Table 2 for detailed examples).

The qualitative evidence highlighted potential sources of bias in the assessment of PA that might have resulted in an overestimation of the increase in recreational PA after retirement in the quantitative review. For example, retirees associated PA with recreational PA but also with a wide range of domestic PA. They believed that sufficient PA levels could be met through activities of daily living. Consequently, quantitative studies that measured recreational PA without clearly defining it might have led to over-reporting. These and other challenges of measuring PA in older adults (e.g. regarding a preference for moderate intensity PA, or the risk of recall bias) have been described in detail in the literature [37-39]. Future studies should clearly define PA, should consider the use of questionnaires specifically designed for older adults [40-43] and should be complemented by studies involving more precise objective assessment of PA. While the contribution of domestic PA to overall PA in old age has been highlighted previously [43-45], there is a lack of evidence on the potential health benefits of domestic PA that should be addressed in future research. Interventions should aim to raise awareness of desirable PA levels for retired individuals and how these can be achieved.

#### Quantitative review finding: the effect of SES and the decrease in PA in retirees from lower occupational groups

Though limited by the small number of qualitative studies [27,31] that included participants from different socioeconomic backgrounds, the available qualitative evidence may help to explain the observed decrease in PA among retirees from lower occupational groups found in the quantitative review. For retired manual workers it was important to remain busy with activities that they perceived as valuable after the transition to retirement. Making additional time for recreational PA was regarded as both impossible and undesirable. Ekerdt’s theory of the ‘busy ethic’ [46] might help to explain this strong urge to stay busy in retirement. According to this theory many retirees shift the eagerness and vigour previously allocated to their work to other purposeful activities in the wish to create continuity between work and retirement. This new busyness might also help to morally justify and defend the concept of retirement to a generation for whom daily manual work had always been existential. Future research should aim to better understand barriers to PA in retirees from lower occupational groups. Interventions promoting purposeful PA (e.g. through working on community projects) and activities that can be integrated into busy lifestyles (e.g. several short bouts over the day) might be more acceptable to, and therefore more effective among, retirees from lower occupational groups.

### Strengths and limitations

This is one of the first studies to have adapted the parallel synthesis approach [16] to the integration of findings from a synthesis of qualitative evidence with those of a synthesis of observational quantitative evidence. Using the qualitative studies to explain and interpret the findings from the quantitative synthesis allowed a better understanding of the quantitative results, raised questions about the validity of the quantitative findings and identified potential avenues for future research and interventions. To minimise publication and language bias, the review was based on a comprehensive literature search including grey research literature and included studies in different languages and from different countries. However, the qualitative evidence is limited by the small number of studies available and the limited socioeconomic diversity of study participants, who were mostly from relatively affluent backgrounds. This limits the robustness of the findings and allowed for only a tentative development of the overarching qualitative themes. Furthermore, there were several challenges involved in comparing and integrating the findings of a qualitative synthesis with those of an existing quantitative review. These included the different scopes and aims of each review (although both examined PA behaviour across the transition to old-age retirement), the different geographical settings of the primary studies (most quantitative studies were set in the United States, whereas the qualitative studies were located in a variety of countries and settings), and the different approaches used to assess PA (all the quantitative studies employed structured assessment tools, whereas PA measurement was less structured in most of the qualitative studies).

## Conclusions

Our findings suggest that adults might increase their recreational PA participation after retirement to improve their health and well-being, to continue lifelong PA patterns, and because PA enables them to establish new daily routines and offers opportunities for social interactions (for women) and personal challenges (for men). PA might decrease among retirees from lower occupational groups because of a lack of time and a perceived low personal value of recreational PA. Future studies should examine the long-term development of recreational PA after the transition to retirement, the broader benefits of PA in retirement and barriers to PA in retirees from lower occupational groups. Interventions should promote multiple benefits of PA, to address different needs and to encourage maintenance of PA after retirement, and address the views and needs of those retiring from manual occupations.

## Competing interests

The authors declare that they have no competing interests.

## Authors’ contributions

IB and DO designed the study. IB conducted the literature search and selected the studies for inclusion. IB and CG contributed equally to the quality appraisal, data extraction and evidence synthesis. All authors contributed to the interpretation of the data and the writing of the manuscript and approved the final version.
